# Maximal Heart Rate for Swimmers

**DOI:** 10.3390/sports7110235

**Published:** 2019-11-12

**Authors:** Bjørn Harald Olstad, Veronica Bjørlykke, Daniela Schäfer Olstad

**Affiliations:** 1 Department of Physical Performance, Norwegian School of Sport Sciences, Oslo 0863, Norway; 2 Polar Electro Oy, Kempele 90440, Finland

**Keywords:** front crawl, running, physiology, training monitoring, training load, training intensity, step-test, sprint, middle distance, athletes

## Abstract

The main purpose of this study was to identify whether a different protocol to achieve maximal heart rate should be used in sprinters when compared to middle-distance swimmers. As incorporating running training into swim training is gaining increased popularity, a secondary aim was to determine the difference in maximal heart rate between front crawl swimming and running among elite swimmers. Twelve elite swimmers (4 female and 8 male, 7 sprinters and 5 middle-distance, age 18.8 years and body mass index 22.9 kg/m^2^) swam three different maximal heart rate protocols using a 50 m, 100 m and 200 m step-test protocol followed by a maximal heart rate test in running. There were no differences in maximal heart rate between sprinters and middle-distance swimmers in each of the swimming protocols or between land and water (all *p* ≥ 0.05). There were no significant differences in maximal heart rate beats-per-minute (bpm) between the 200 m (mean ± SD; 192.0 ± 6.9 bpm), 100 m (190.8 ± 8.3 bpm) or 50 m protocol (191.9 ± 8.4 bpm). Maximal heart rate was 6.7 ± 5.3 bpm lower for swimming compared to running (199.9 ± 8.9 bpm for running; *p* = 0.015). We conclude that all reported step-test protocols were suitable for achieving maximal heart rate during front crawl swimming and suggest that no separate protocol is needed for swimmers specialized on sprint or middle-distance. Further, we suggest conducting sport-specific maximal heart rate tests for different sports that are targeted to improve the aerobic capacity among the elite swimmers of today.

## 1. Introduction

Training planning and implementation consists of three key components: frequency, duration and intensity. While frequency and duration are simple to monitor, monitoring training intensity is more difficult. Training intensity can be determined by ventilatory and metabolic thresholds [1]. Using gas analyzers to measure ventilatory threshold zones is a non-invasive technique, but contains disadvantages related to cost and equipment constraints, which makes it difficult to apply and operate in training conditions, especially during swimming [1]. Portable lactate analyzers can allow for a more practical and convenient assessment, even though they are a slightly invasive method. However, measuring lactate can be expensive and time consuming for monitoring daily intensity. It is, therefore, crucial that other less complex and inexpensive methods can be used to monitor the daily training intensity [2], such as speed, rating of perceived exertion (RPE) and heart rate (HR) [3].

To assess an optimal way of measuring training intensity, it is important to find the right balance between the degree of validity and practical application [4]. In swimming, speed is often used as a criterion. Many coaches apply a variety of tests such as T-30, V4, various step-tests and critical swim speed, often in combination with lactate to predict the speed for different intensity zones during training [5]. The limitation of such approaches is that such tests are usually not performed regularly enough to capture daily changes, such as signs of sickness, fatigue and overreaching [4]. RPE is another method that can define the daily training intensity and load. However, this is a subjective method and does not always correspond to the actual training intensity described by the coach [6]. Monitoring HR during exercise is particularly useful in endurance training [7]. It is an objective method that is simple, affordable, easily accessible and can be recorded continuously during training. New technologies, such as the Polar OH1 optical HR sensor or Polar H10 HR sensor with Polar Pro strap, transmit data via Bluetooth to the Polar Team app, making it possible for the coach to track the HR-based intensity of all swimmers on a tablet live during training. This makes the use of HR for intensity monitoring in swimming more attractive. Based on measurements from maximum HR (maxHR) tests, HR zones can be calculated and used to indicate different intensity zones [4]. However, when intensity zones are derived from maxHR, it is important to consider that maxHR might vary between sports on land and in the water and between different types of athletes [8,9,10,11,12]. If intensity zones are calculated based on maxHR values on land or from different formulas predicting a person’s maxHR, this may result in the actual training being carried out with the wrong workload and in the failure to achieve the desired training benefit.

Knowledge about the right protocol to achieve maxHR in swimming is sparse, most likely due to the lack of valid technology to measure HR in the water in the past. The traditional approach for evaluating the physiology of competitive swimmers has been centered on aerobic fitness through similar protocols. However, when interpreting these results, it is important to encounter the underlying type of swimmer (sprint, middle-distance or distance) [13]. Therefore, the three swimming step-test protocols in this study were designed with differences in intervals and total distance, with the hypothesis that it may possibly benefit different types of swimmers. To our knowledge, nobody has yet investigated whether a different protocol to achieve maxHR should be used for swimmers specialized on the different distances.

Moreover, it is getting more popular to incorporate different exercise modes into the swimming workout routines to gain higher cardiovascular fitness without the overuse of swimming specific muscles. For example, the Norwegian swimming federation recommends swimmers to include two to four running sessions per week prior to the competitive period [14]. It is well known that maxHR is different between various sports. Most studies that reported maxHR in swimming were conducted with either triathletes or fitness swimmers. Two studies investigated the difference in maxHR between swimming (mix of strokes) and running with the same population of elite swimmers through VO_2max_ testing. They found maxHR to be 15 beats-per-minute (bpm) lower during swimming (boys 184 ± 11; girls 186 ± 10 bpm) compared to running (boys 199 ± 10; girls 201 ± 7 bpm) [15,16]. As these studies were conducted in the 70s, and it remains unknown how much of a difference in maxHR is expected in modern elite swimmers that appear to have a higher muscle mass and how different maxHR is between cycling, running and front crawl swimming. However, knowing the difference in maxHR between front crawl swimming and other sports used regularly in training is essential for monitoring the right intensity among competitive swimmers.

The main purpose of this study was, therefore, to identify whether a different protocol to achieve maxHR should be used in sprinters than in middle-distance swimmers, and secondly, to determine the difference in maxHR between front crawl swimming and running/cycling among elite swimmers. We hypothesized that a different protocol should be used to asses maxHR in sprinters vs. middle-distance swimmers, and that maxHR would be lower in front crawl swimming compared to running/cycling.

## 2. Materials and Methods

### 2.1. Participants

Twelve elite swimmers at the national and international competition level (four women and eight men) participated voluntarily. Seven were classified as sprinters and five as middle-distance swimmers. Sprinters had the highest Fédération Internationale de Natation (FINA) points in either a 50 m or 100 m event, and middle-distance swimmers had their highest FINA-points in either a 200 m or 400 m event. Physical characteristics and FINA-points of the participants are shown in Table 1. One swimmer was previously diagnosed with asthma and uses Airomir in conjunction with training and competitions. All other participants were healthy according to the self-declaration form including questions regarding recent illnesses and injuries and own medical and family history.

The participants were given detailed verbal and written explanation of the aims, procedures and any risks involved in the investigation. They completed a health history questionnaire including details on training activity levels prior to participation. Eligible participants or the legal guardian then provided written informed consent to participate in the study. All procedures were performed in accordance with the recommendations of the Declaration of Helsinki. This study was approved by the local ethics committee and the National Data Protection Agency.

Prior to data collection, all participants were familiarized with the test procedures and equipment and were verbally encouraged to perform to exhaustion during all tests. Prior to every protocol, participants were instructed to abandon hard physical exercise over the last 48 h.

Two participants failed to complete all protocols in the study (one sprinter did not carry out the 50 m step-test in the water due to illness, and two middle-distance swimmers did not complete the land protocol due to illness).

### 2.2. Procedures

Participants performed three front crawl step-tests (50 m, 100 m and 200 m) in a randomized order in the same 25 m indoor swimming pool with six lanes and a water temperature of about 27 °C. Participants were blinded from their test results until all protocols were completed. Measurements took place between 05:00 and 08:00 PM with approximately one week apart between each protocol. A 2000 m standardized warm-up was performed prior to each test protocol (Table 2). Participants were divided into three lanes (four in each lane), where three highly experienced swim coaches with high knowledge of the participants training background were responsible for organizing the lanes, following the participants with split times and giving continuous feedback on the pace. All three coaches had more than five years of experience in coaching age-group swimmers to national and international swimmers and had completed the national coaching education program.

Three weeks after the last swimming protocol, participants performed a maxHR test on land between 05:00 and 08:00 PM. They performed a running protocol on a motorized treadmill (Technogym, Excite run 600, Cesena, Italy). If they were not able to run due to knee pain, they performed a cycle protocol on an ergometer bike (Technogym, Excite Bike, Cesena, Italy) instead. Cycling elicits a different movement activity compared to running. In triathletes, the maxHR observed in cycling is often lower by 6–10 beats/min than that obtained during running [17]. However, in contrast, there is also evidence suggesting that maxHR is similar between cycling and running [17]. We do not know if maxHR in cycling is different from running in elite swimmers, but we chose cycling as alternative to running, as cycling appears to be a common alternative to running for supplementing swim training. Prior to the experiment, participants performed a standardized warm-up on the treadmill [18]. In order to avoid muscular fatigue, the warm-up protocol was slightly modified [19]. It consisted of 15 min with 5 min of walking with a HR under 150 bpm followed by 10 min with a progressive increase in velocity until completion when HR was approximately 20 bpm under the expected maxHR based on previous testing results. A corresponding warm-up protocol was carried out on the ergometer bike.

### 2.3. Test Battery 

#### 2.3.1. Step-Test Protocols in Swimming

The 50 m step-test protocol was adapted based on common practice for achieving maximum aerobic training [5], and is presented in Table 3. The 100 m step-test protocol is shown in Table 4 [20]. The 200 m step-test protocol was based on individual target times that were calculated in advance based on the participants personal best (PB) time in the 200 m front crawl (Table 5) [21].

#### 2.3.2. Test Protocol for Land Measurements

A velocity and inclination ramp test was performed on the treadmill with a pre-defined increase in speed (2 min at 8.5 km·h^−1^, and then a rise of 1.5 km·h^−1^ every 30 s up to 14.5 km·h^−1^) and thereafter inclination (0.5° every 30 s) [22]. The three participants unable to run performed a seated step-test protocol on an ergometer bike. The cycling step-test began with a workload of 60 watts and then increased by 30 watts every minute until exhaustion [23]. The protocols were selected based on the predicted abilities of the participants. Moreover, a steady increase in load during an incremental test (every 30–60 s) allows the cardiopulmonary system to respond gradually [24].

### 2.4. Data Collection and Analysis

Pilot tests were conducted prior to the experiment to ensure familiarization with the technology. During all protocols, participants were equipped with two HR monitors that recorded HR continuously. The Polar H10 HR sensor with the Polar Pro strap (Polar Electro Oy, Kempele, Finland) was worn around the chest. During the swimming protocols, this was placed underneath a swimsuit. Male swimmers wore a suit covering their chest to avoid the chest strap from sliding down during the push-off from the wall. Based on the participants’ feedback, we assume that wearing the suit had a negligible effect on technique and performance of the swimmers. A Polar OH1 optical HR sensor (Polar Electro Oy, Kempele, Finland) was placed at the temple underneath the swim cap through a customized headband made from an old HR chest strap. Second by second HR was used for analysis.

HR data were stored in the internal memory of the Polar H10 HR sensor. Polar Beat version 2.4.5 for Android (Polar Electro Oy, Kempele, Finland) was installed on each participant’s cellular phone for transferring HR data from H10 through Bluetooth. After the completion of each test protocol, data were uploaded, and HR was analyzed through Polar Flow (Polar Electro Oy, Kempele, Finland). In addition, HR from H10 and OH1 were live transmitted throughout the protocols through Bluetooth to a tablet (iPad 4, Apple, Inc., Cupertino, CA, USA) where the participants were continuously monitored by the researchers using Polar Team app version 1.3 (Polar Electro Oy, Kempele, Finland) to ensure good data quality and control for effort. After inspecting the data for possible abnormalities, maxHR was defined as the highest registered measurement during each protocol [25].

IBM SPSS^®^ Statistics v24.0 (IBM^®^ Corporation, Armonk, NY, USA) and Microsoft Excel 2010 (Microsoft^®^ software, Microsoft Corporation, Redmond, WA, USA) were used for all statistical computations. A Shapiro–Wilk analysis was used to test for normal distribution of the data. Descriptive analysis is reported as mean and one standard deviation. Due to the small sample size, non-parametric statistics were used [26]. Mann–Whitney Tests were used to assess whether there was a difference between sprinters and middle-distance swimmers for maxHR in the water or maxHR on land or any of the three different swimming protocols. Kruskal–Wallis Tests were used to assess the effect of the different swim protocols on maxHR. Wilcoxon Signed Ranks Tests were used to assess statistical differences between maxHR in the water and on land. The significance level was *p* < 0.05.

## 3. Results

One out of 12 swimmers (male sprinter) was not able to complete the 50 m swimming protocol and the land exercise as he got sick. Another swimmer (female sprinter) completed all swimming but not the land protocol due to sickness. One swimmer was not able to complete the 50 m swimming protocol due to an asthma attack. Seven out of 10 swimmers completed the land protocol running on the treadmill, and 3 were bicycling on the ergometer due to knee pain (all sprinters). Overall results are shown in Table 6.

**Figure 1:**
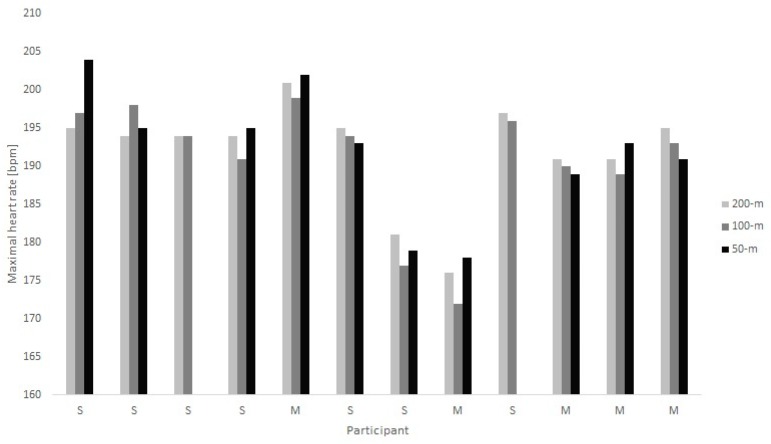
Maximal heart rate achieved in the 50 m, 100 m and 200 m protocol. There were no significant differences between sprinters and middle-distance swimmers in any of the protocols. S = Sprinter, M = Middle-distance swimmer.

**Figure 2:**
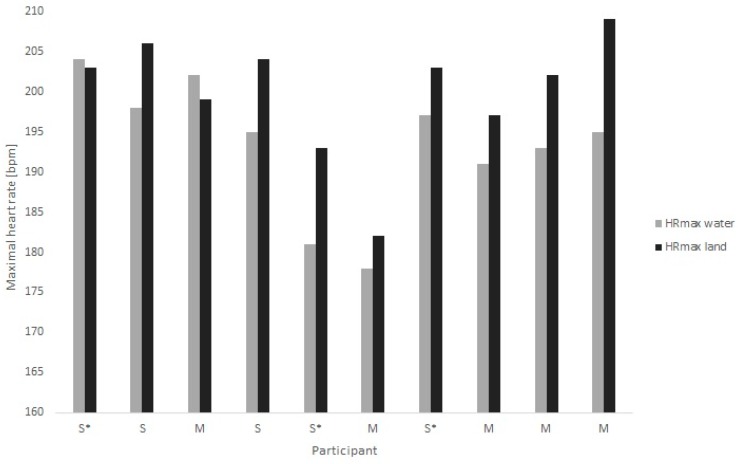
Maximal heart rate achieved in the water compared to maximal heart rate achieved on land. Most swimmers achieved higher maximal heart rate on land compared to water. S = Sprinter, M = Middle-distance swimmers, HRmax = maximal heart rate, and * = the participant performed the land protocol on the bicycle ergometer instead of the running treadmill.

Individual results for the swimming protocols are shown in Figure 1 and in the comparison between water and land in Figure 2.

Mann–Whitney Tests showed no differences in maxHR between sprinters and middle-distance swimmers in each of the swimming protocols or between maxHR on land or in the water (all *p* ≥ 0.05). Kruskal–Wallis Tests showed no significant differences in maxHR between any of the swimming protocols. Presented *p*-values for the Kruskal–Wallis Test in Table 6 were calculated including all participants. In order to make sure that participants not completing all swimming protocols did not alter the results, *p*-values for the Kruskal–Wallis Test were also calculated separately only including participants that completed all swimming protocols. Results were similar (*p* = 0.331 for sprinters, *p* = 1.000 for middle distance and *p* = 0.464 for all swimmers together). Five out of 12 participants showed the highest maxHR in the 200 m protocol, one out of 12 in the 100 m protocol and five out of 10 in the 50 m protocol. One participant had an equal highest maxHR in the 200 m and 100 m protocol.

In the 200 m step-test protocol, 10 swimmers reached their maxHR during the last (=7th) 200 m, while 2 swimmers reached it during the 6th 200 m. In the 100 m step-test protocol, 6 swimmers reached maxHR during the last 100 m in the 3 × 100 m, 5 swimmers in the last 100 m after 3 × (3 × 100 m) and one swimmer in the last 100 m in the second round of the 3 × 100 m. In the 50 m step-test protocol, 4 swimmers reached maxHR in the last 50 m of the 8 × 50 m, 4 swimmers in the last 100 m after 4 × (8 × 50 m), one swimmer in the 7th 50 m in the last 8 × 50 m and one swimmer in the 5th 50 m in the last 8 × 50 m.

Land protocols (running and cycling together) showed a significantly higher maxHR (199.8 ± 7.7 bpm) compared to highest measured maxHR in the water (193.6 ± 7.5 bpm; *p* = 0.012). Compared to the highest measured maxHR in swimming (193.6 ± 7.5 bpm), maxHR was 6.7 ± 5.3 bpm higher in running and 5.7 ± 6.5 bmp in cycling, respectively. When comparing maxHR in the water to running and cycling separately, only running showed a significantly higher maxHR (*p* = 0.028). Two participants showed a higher maxHR in the water compared to land, whereof one doing the running and one the cycling protocol.

## 4. Discussion

We found no differences in maxHR between sprinters and middle-distance swimmers in each of the swimming protocols or between land and water (all ≥ 0.05). There were no significant differences in maxHR between the 200 m, 100 m or 50 m protocol, while running showed significantly higher maxHR than swimming.

### 4.1. Front Crawl Swimming Step-Test Protocol Versus Type of Swimmer

None of the three selected swimming protocols seems to be better suited to sprinters or middle-distance front crawl swimmers. Moreover, the type of protocol did not have an effect on maxHR. To our knowledge, no such analysis has been performed previously in swimming or any other sport with sprint and middle-distances, such as running. We hypothesized that the interval distance of 200 m in the 200 m step-test protocol may have been experienced too long to keep required maximal effort compared to what sprinters are used to from their daily training. This lack of training of the 200 m protocol could have caused difficulties with correctly pacing the maximal effort. This could have led to mental and physiological fatigue, influencing the results. In contrast, we hypothesized that middle-distance swimmers would experience the 50 m and 100 m distance step-test protocols to be too short to reach maximal effort. It could, for example, be that they were physically not able to swim at a pace that would equalize maximal effort for a shorter distance. However, it seems like the protocols were robust towards reaching maximal effort independent of the specialized distances of the swimmers. As there were no significant difference in maxHR between the tests, we suggest that the maxHR test should be selected according to personal preference. However, as only 1 out of 10 participants reached maxHR in the 100 m step-test protocol, and the average maxHR in the 100 m protocol was 1.1 bpm lower than in the 50 m and 1.2 bpm lower than in the 200 m protocol, we suggest that selecting the 50 m or 200 m protocol over the 100 m protocol may give a slightly higher maxHR.

### 4.2. Differences in MaxHR on Land and in the Water

MaxHR was lower during front crawl swimming compared to land measurements from running and were, on average, 6.7 ± 5.3 bpm (*p* = 0.015) and ergometer cycling was 5.7 ± 6.5 bpm (*p* > 0.05) among elite swimmers. Thus, elite swimmers of today still have significantly lower maxHR in water than on land, and these values were substantially lower than the 15 bpm difference recorded between swimming and running in the 70s [15,16]. Possible reasons for this could be that the previous studies did not measure front crawl, but a mix of swimming strokes as well as the likely higher muscle mass in today’s elite swimmers.

Several studies have found larger differences in maxHR between swimming, running and cycling among different types of athletes. For triathletes and fitness swimmers, the maxHR was 9.0–14 bpm lower during swimming than for running and cycling [9,10,12,27,28]. A possible explanation of these differences can be related to the sport specific training adaptations. The training of elite swimmers is specifically aimed at improving the swimming performance, and may lead to better adaptations to the muscle work required from the arms and upper body as well as mastering the swimming technique at a more economical level [29]. Triathletes and fitness swimmers may experience muscular fatigue in the upper body earlier than elite swimmers and before they might have reached their maxHR, while elite swimmers might lack the sport specific endurance during running and cycling. It is also thought that the difference in training volumes between triathletes and swimmers [27], and the function of acute physiological responses combined with the training specificity of elite swimmers, can influence the results and differences in maxHR [12].

Several studies have tried to explain why maxHR might be lower during swimming compared to, for example, running and cycling on land. One possible reason could be that swimming is carried out in a horizontal position [15]. This can cause a reduced blood flow to the lower extremities through a lower arterial pressure compared to when gravity is acting on the body in a vertical position [11]. HR at rest was also found to be lower in supine compared to seated and standing body position on land [9,30]. In front crawl swimming, the arms and upper body are mainly responsible for generating propulsion. The difference in the proportion of active muscle mass in swimming compared to running and cycling could, therefore, affect the requirements for the cardiovascular system and account for the differences in maxHR between swimming and cycling or running [29,31]. Buoyancy in the water also compensates gravity and, therefore, a minimal part of the muscle mass is required to support the body in the water, preventing the activation of large muscle mass in the lower extremity when compared to running [28]. A study comparing maximal oxygen consumption with varying arm and leg exercises found HR to be higher during running when compared to exercises where both the arms and legs were separately active [8]. MaxHR was also reported to be lower during exercise with predominantly upper body work compared to running in highly-trained cross-country skiers [32]. Also, together with the large proportion of the blood flow occurring in the upper body, during front crawl swimming the heart will increase the stroke volume [33]. Other possible reasons for the difference in maxHR on land and in the water have been presented as: the ability to breathe is limited in water, external pressure is increased and the thermal conductivity is higher in water than in the air [28].

DiCarlo et al. [9] proposed to calculate maxHR in swimming by subtracting a given absolute number (12 bpm) obtained from treadmill running or predicted maxHR. However, because of the high SD in the maxHR difference between swimming and running (6.7 ± 5.3 bpm) or cycling (5.7 ± 6.5) protocols, we recommend to test the maxHR in each individual swimmer for sports regularly used in swim training to improve aerobic capacity.

### 4.3. Limitations

Instead of lane-line pacing through lights, swimming pace was controlled by the dedicated swim coach, which could have led to small deviations in pace during the swimming protocols. The participants performed only one repetition of maxHR on land, while three in the water. The protocols for running and cycling were chosen based on established protocols from previous studies [22,23]. Whether those protocols are successful in reaching maxHR in running and cycling among elite swimmers remains unknown. However, as the HR plateau was reached at the end of the test protocols, we assume that maxHR was successfully reached. The small sample size is a further limitation of this study.

### 4.4. Practical Implications

The findings from this study help improve monitoring and controlling intensity using HR as a tool during the daily swim training. Practitioners should focus on the following key points while using HR as a tool for monitoring swimming intensity:

- All step-test protocols described in this study were suitable for achieving maxHR during front crawl swimming.
- When training intensity is monitored with HR, it is important to know the sport-specific maxHR. In swimming, it is mostly important to determine the right maxHR for swimming. However, when land training such as running or cycling are incorporated into swim training, it is important to test maxHR separately for these sports.
- As the difference between maxHR on land and in the water showed a high SD, it is essential to perform a land maxHR test in addition to maxHR test for swimming, instead of using the average difference found in this study to ensure correct intensity guidance also during land training. Note that running and cycling was performed on an ergometer in the lab in this study. Results might be different in the field due to slightly different movement patterns and environmental effects including temperature and humidity.

## 5. Conclusions

We conclude that all reported step-test protocols were suitable for achieving maxHR during front crawl swimming and suggest that no separate protocol is needed for swimmers specialized for sprint or middle-distance. MaxHR was lower for elite swimmers during front crawl swimming compared to running on a treadmill or ergometer bicycling. This shows the importance of a sport specific maxHR test for swimmers to use HR as a parameter for intensity control and monitoring training load.

The high individual variation in the difference in maxHR between front crawl swimming compared to running or cycling highlights the importance of sport-specific maxHR tests for all type of aerobic exercises used by elite swimmers.

## Figures and Tables

**Table 1:** Characteristics of participants mean ± SD.

Athlete Type	FINA Points *	Gender (n)	Age (year)	Body Mass (kg)	Height (m)
Sprinter	698.4 ± 33.6	3 women	17.3 ± 2.9	60.7 ± 5.1	1.68 ± 0.1
		4 men	18.3 ± 1.7	80.0 ± 5.2	1.85 ± 0.1
Middle-distance	678.4 ± 35.5	1 woman	18	72	1.78
		4 men	20.5 ± 3.3	79.8 ± 10.2	1.85 ± 0.1

* The highest number of Fédération internationale de natation (FINA) points for each swimmer, regardless of distance and pool length.

**Table 2:** Standardized warm-up for the three test protocols in water.

Distance (m)	Swimming Stroke	Intensity Zone	Start Time (min)
400	Crawl	1–2	6:00
3 × 200	Crawl	1–3	3:00
8 × 50	Drill/crawl	1–2	1:00
4 × 100	Pace/crawl	3–5	1:50
200	Choice	1	None

**Table 3:** Test protocol for the 50 m step-test.

Distance (m)	Step (#)	Repetition (#)	Start Time (s)	Intensity Zone
50	1	8	50	4—Threshold pace
50	2	8	55	4–5
50	3	8	60	5—VO_2max_ pace
50	4	8	60	5–6
100	5	1	None	Maximal effort

1–2 min of passive rest between each step in the protocol. All repetitions began with start in the water. Intensity zones are based on the national intensity scale for endurance sports (https://www.olympiatoppen.no/fagstoff/utholdenhet/oltsintensitetsskala/page594.html#).

**Table 4:** Test protocol for the 100 m step-test.

Distance (m)	Step (#)	Repetition (#)	Start Time (min)	Intensity (I-zone)
100	1	3	1:40	3—Aerobic pace
200		1	4:00	1
100	2	3	1:50	4—Threshold pace
200		1	4:00	1
100	3	3	2:00	5—VO_2max_ pace
200		1	4:00	1
100	4	1	None	Maximal effort

All repetitions began with start in the water. Intensity zones are based on the national intensity scale for endurance sports (https://www.olympiatoppen.no/fagstoff/utholdenhet/oltsintensitetsskala/page594.html#).

**Table 5:** Test protocol for the 200 m step-test.

Distance (m)	Step (#)	Repetition (#)	Rest (min)	Intensity (PB+)
200	1	1	1:15	35 s
200	2	1	1:15	30 s
200	3	1	1:15	25 s
200	4	1	1:15	20 s
200	5	1	1:15	15 s
200	6	1	1:15	10 s
200	7	1	1:15	Maximal effort

Intensity was calculated from the participant’s personal best time (PB) on the distance. All repetitions began with start in the water.

**Table 6:** Overall results.

Swimmer Type	200 m Step-Test (mean ± SD)	100 m Step-Test (mean ± SD)	50 m Step-Test (mean ± SD)	MaxHR Water (mean ± SD)	MaxHR Land (mean ± SD)	Effect of Swim Protocol on MaxHR (Kruskal–Wallis Test)	Statistical Difference between Land and Water (Wilcoxon Signed Ranks Tests)
Sprinters	192.9 ± 5.3 (*n* = 7)	192.4 ± 7.2 (*n* = 7)	193.2 ± 9.0 (*n* = 5)	194.9 ± 7.0 (*n* = 7)	201.8 ± 5.1 (*n* = 5)	*p* = 0.265	*p* = 0.080
Middle-distance swimmers	190.8 ± 9.2 (*n* = 5)	188.6 ± 10.1 (*n* = 5)	190.6 ± 8.6 (*n* = 5)	191.8 ± 8.8 (*n* = 5)	197.8 ± 9.9 (*n* = 5)	*p* = 1.000	*p* = 0.080
All swimmers	192 ± 6.9 (*n* = 12)	190.8 ± 8.3 (*n* = 12)	191.9 ± 8.4 (*n* = 10)	193.6 ± 7.5 (*n* = 12)	199.8 ± 7.7 (*n* = 10)	*p* = 0.467	*p* = 0.012
Effect of sprinters vs. middle-distance swimmers on maxHR (Mann–Whitney Test)	*p* = 0.564	*p* = 0.290	*p* = 0.293	*p* = 0.288	*p* = 0.346	NA	NA

Abbreviations: maxHR = maximal heart rate; SD = standard deviation; *n* = number of participants; NA = not applicable.
